# Optimization of methods for the accurate characterization of whole blood neutrophils

**DOI:** 10.1038/s41598-022-07455-2

**Published:** 2022-03-07

**Authors:** Ashley N. Connelly, Richard P. H. Huijbregts, Harish C. Pal, Valeriya Kuznetsova, Marcus D. Davis, Krystle L. Ong, Christian X. Fay, Morgan E. Greene, Edgar T. Overton, Zdenek Hel

**Affiliations:** 1 Department of Pathology, University of Alabama at Birmingham, Birmingham, AL USA; 2 Department of Microbiology, University of Alabama at Birmingham, Birmingham, AL USA; 3 Center for AIDS Research, University of Alabama at Birmingham, Birmingham, AL USA; 4 Division of Infectious Disease, University of Alabama at Birmingham, Birmingham, AL USA

**Keywords:** Immunology, Innate immune cells, Innate immunity

## Abstract

Neutrophils are the most abundant circulating leukocyte population with critical roles in immune defense, regulation of innate and adaptive immune systems, and disease pathogenesis. Our progress in understanding precise mechanisms of neutrophil activation, recruitment, and function has been hampered by the lack of optimized and standardized methods for the characterization and phenotyping of this readily activated population. By comparing eight methods of neutrophil characterization, we demonstrate that the level of neutrophil activation and degranulation is associated with specific experimental conditions and the number and type of manipulation steps employed. Staining whole blood at 4 °C and removal of remaining unbound antibodies prior to one-step fixation and red blood cell lysis minimizes neutrophil activation, decreases phenotypic alterations during processing, and prevents nonspecific antibody binding. The effects of anticoagulants used for collection, processing delays, and time and temperature during sample analysis on neutrophil phenotype are addressed. The presented data provide a foundation for higher quality standards of neutrophil characterization improving consistency and reproducibility among studies.

## Introduction

Neutrophils constitute 50–70% of circulating leukocytes and are increasingly recognized as a heterogeneous population with critical roles in immune regulation^1–3^ and disease pathogenesis^4,5^. Comprehensive characterization of neutrophils and neutrophil subsets is hampered by their inherent ex vivo instability, their tendency to form multiplets with other cell types, and a high level of nonspecific background staining likely due to the exposure of cationic proteins during activation. Neutrophils are known to become rapidly activated by many common methods of preparation including red blood cell (RBC) sedimentation^6^, RBC lysis^7^, and density gradient centrifugation^8^. Several neutrophil subpopulations have recently been described in homeostatic and pathological conditions based on phenotypic, transcriptional, and functional properties^2,4,5,9–14^. Low-frequency subpopulations are typically identified based on the levels of specific surface proteins that may be altered by activation during preparation, making accurate characterization of neutrophil subsets challenging. Despite the discrepancies between studies, prior publications have not systematically compared characterization methods to determine approaches minimizing neutrophil activation. Currently used methods for neutrophil characterization often rely on combinations of density gradient centrifugation and RBC removal by sedimentation and/or lysis^15–21^. Individual studies utilize specific preparation methods, type of anticoagulant for blood collection, and timeframes for sample processing resulting in reports with varying levels of activation and artifactual changes in phenotype. This presents a significant obstacle for accurate characterization of neutrophil phenotype and complicates comparison of results among studies, impeding progress in the field.

In clinical research, blood collection often occurs at locations distant from the site of analysis, making delays in sample processing unavoidable. This is especially problematic for neutrophils since they cannot be cryopreserved for delayed analysis and must be processed fresh. Several studies investigated the functional capacity of neutrophils following 24–72 h of incubation, finding that with time neutrophils have reduced capacity for phagocytosis^22,23^, bacterial killing^22,24^, chemotaxis^22,24,25^, random movement^25^, and oxidative burst^25,26^. However, the phenotypic changes that occur due to delayed processing have yet to be described. Other key variables differing among neutrophil studies include staining temperature and type of anticoagulant used for blood collection that were shown to influence neutrophil recovery and response to stimulation^27^. Current knowledge regarding the effects of these factors on neutrophil phenotype is limited.

Here we determine the individual effects of delayed sample processing, staining temperature, and type of anticoagulant employed on neutrophil phenotype by directly comparing eight common methods of neutrophil characterization in relation to neutrophil recovery, activation, and multiplet formation. Our data indicate that staining whole blood at 4 °C within 3 h of blood draw, removal of unbound antibodies to minimize nonspecific antibody binding, followed by treatment with One-step Fixation and RBC Lysis buffer (hereafter referred to as Fix/lyse buffer) minimizes neutrophil activation and multiplet formation while maximizing neutrophil recovery.

## Results

### Comparison of common methods of neutrophil characterization

**Figure 1:**
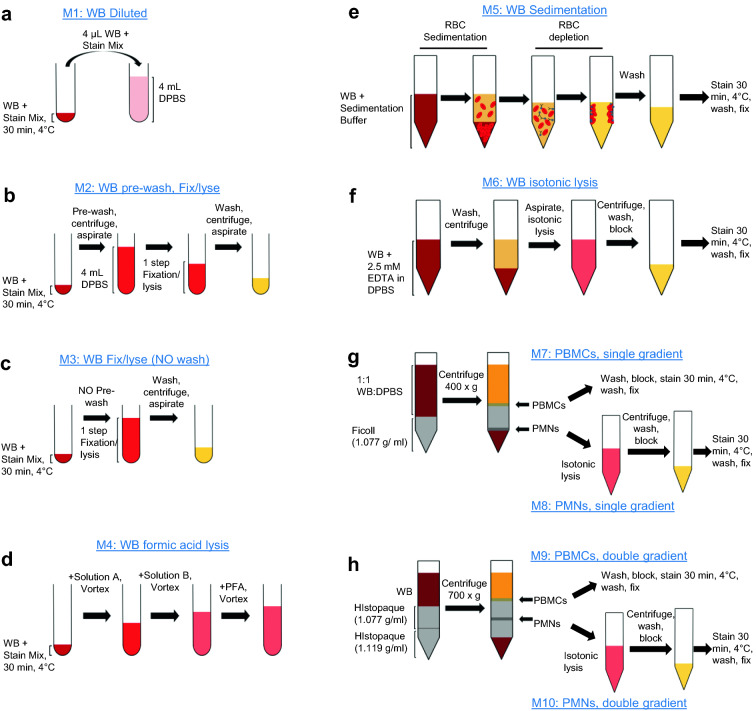
Neutrophil characterization methods. Schematic representations of tested methods. (**a**) Method M1; WB staining and dilution. (**b**) Method M2; whole blood staining followed by pre-washing antibodies and 1-step fixation and RBC lysis. (**c**) Method M3; WB staining followed by 1-step fixation and RBC lysis without pre-washing antibodies. (**d**) Method M4; WB staining followed by formic acid lysis and PFA fixation. (**e**) Method M5; RBC sedimentation of WB, followed by RBC depletion by magnetic beads, staining, washing, and fixation. (**f**) Method M6; isotonic lysis, followed by washing, staining, washing, and fixation. (**g**) Methods M7 and M8; WB dilution and single layer discontinuous density gradient centrifugation using Ficoll (1.077 g/ml). PBMCs localize above the gradient and are processed as M7 (top part of panel): cells are washed, blocked, stained, washed, and fixed. PMNs localize below the gradient and are processed as M8 (bottom part of panel): after PBMC collection, the remaining Ficoll is removed, cells are isotonically lysed, washed, blocked, stained, washed, and fixed. (**h**) Methods M9 and M10; WB is layered on a double layer discontinuous density gradient of histopaque (top layer—1.077 g/ml and bottom layer—1.119 g/ml) and centrifuged. PBMCs localize at the intersection of the plasma and the top gradient and are processed as M9 (top part of panel): cells are collected, washed, blocked, stained, washed, and fixed. The PMNs localize at the interface between the two gradients and are processed as M10 (bottom part of panel): any remaining upper gradient is removed and cells are isotonically lysed, washed, blocked, stained, washed, and fixed. All samples are analyzed by multiparametric flow cytometry.

Eight commonly used methods of neutrophil characterization, requiring varying numbers of manipulation steps such as RBC depletion, centrifugation, staining, and fixation, were directly compared in parallel settings. Each method is described in detail in the Methods section and depicted in Fig. 1. A summary of the manipulation steps involved in each method is included in Table 1. As a reference for method comparison, the “whole blood diluted” method (M1; Fig. 1a) was utilized. This method is based on a rapid dilution of a small amount of stained whole blood in DPBS at 1:2000 final ratio immediately prior to sample analysis by flow cytometry with acoustic focusing technology without further processing. This method allows for the analysis of neutrophils close to their in vivo state by elimination of most processing steps; however, due to the slow rate of acquisition of cells of interest in the presence of unlysed RBCs, this method is not suitable for routine laboratory use. The dilution method was compared with other methods for neutrophil characterization in whole blood with minimal (methods M2-M4, Fig. 1b–d) or moderate manipulation (methods M5 and M6, Fig. 1e,f) and methods based on gradient separation (methods M7-M10, Fig. 1g,h) analyzing neutrophils from the peripheral blood mononuclear cell (PBMC) (methods M7 and M9) and polymorphonuclear cell (PMN) (methods M8 and M10) layers.

**Table 1:** Manipulation steps during neutrophil characterization.

	M1	M2	M3	M4	M5	M6	M7	M8	M9	M10
Sedimentation					1					
RBC magnetic depletion					1					
Gradient Centrifugation							1	1	1	1
RBC lysis before fixation				1		1		1		1
Wash(es) before stain					1	3	2	3	2	3
Fc block					1	1	1	1	1	1
Stain	1	1	1	1	1	1	1	1	1	1
Pre-wash after stain		1			1	1	1	1	1	1
Fixation		1	1	1	1	1	1	1	1	1
Total manipulation score	1	3	2	3	7	8	7	9	7	9
Method	WB diluted	WB pre-wash, fix/lyse	WB fix/lyse (no wash)	WB formic acid lysis	WB sedimen-tation	WB isotonic lysis	PBMCs (single gradient)	PMNs (single gradient)	PBMCs (double gradient)	PMNs (double gradient)

Summary of the steps performed during neutrophil characterization by each method (M1–M10) quantified as the total number of manipulation steps before fixation.

**Figure 2:**
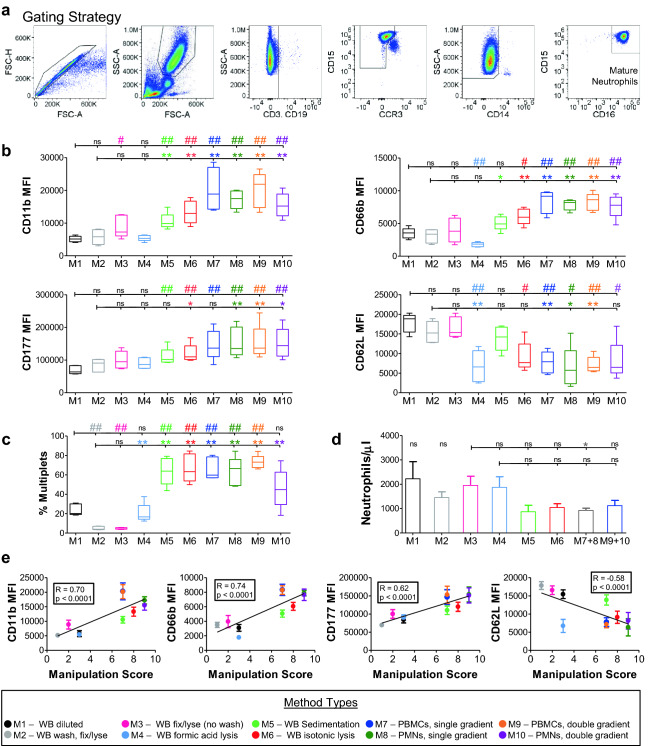
Comparison of methods for neutrophil characterization. (**a**) Strategy for identification of mature neutrophils gated as single cells, SSC and FSC high, and positive for neutrophil markers CD15 and CD16. Negative gates for lineage exclusion were used to identify multiplets with T cells (CD3), B cells (CD19), eosinophils (CCR3), and monocytes (CD14). (**b**) Neutrophil surface levels of indicated proteins following characterization by the methods M1–M10 as defined in Fig. 1 and Methods section. MFI, median fluorescence intensity. Box lines indicate 25th, 50th, and 75th percentiles; whiskers indicate minimum and maximum. (**c**) Percentage of neutrophils forming multiplets during processing using the indicated characterization method. (**d**) Neutrophil recovery per microliter of WB following processing by the indicated method. Bars represent means/SEM. (**b**–**d**) Methods were compared using Mann–Whitney test ^#^*p* < 0.05, ^##^*p* < 0.01 compared to M1 while *p < 0.05, **p < 0.01 compared to M2, n = 5. (**e**) Linear regression analyses of indicated neutrophil surface marker levels and manipulation scores as calculated in Table 1 for each tested method. Points are plotted as means ± SEM. In all cases blood was collected in ACD tubes and stained at 4 °C.

Mature neutrophils were carefully gated as SSC-A^high^ single cells expressing CD15 and CD16 (FcγRIII)^28^. Neutrophils readily form complexes with other cell types, creating neutrophil-leukocyte multiplets (NLMs). To ensure the purity of the analyzed neutrophil population, other cell types and NLMs were eliminated using negative gates for CD3 (T cells), CD19 (B cells), CCR3 (eosinophils), and CD14 (monocytes) (Fig. 2a). The neutrophil phenotype was assessed for alteration of levels of physiologically relevant surface proteins including L-selectin (CD62L) that is shed from the surface of neutrophils upon activation^29,30^, and CD11b and CD66b that are stored in secondary granules of neutrophils and the surface levels of which increase following degranulation^31,32^. Degranulation, determined as an increase of the median fluorescent intensities (MFIs) of CD11b and CD66b, was minimized when methods M2 (WB pre-wash, Fix/lyse), M3 (WB Fix/lyse, no pre-wash), or M4 (WB formic acid lysis), were employed (Fig. 2b). Neutrophil activation, determined as increased CD177^33^ and CD16^28^ and decreased CD62L MFIs, was minimized by methods M2 and M3, (Fig. 2b and Supplementary Fig. S1a). Method M4 exhibited decreased CD62L levels, although CD177 and CD16 remained low following preparation by this method, suggesting minimal neutrophil activation. Methods M5 and M6 demonstrated moderate activation determined as increased CD177 for both methods and decreased CD62L for method M6 (Fig. 2b). Increased neutrophil activation by methods M7-10 (single and double gradient methods) was indicated by a significant downregulation of CD62L and upregulation of CD177 (Fig. 2b). Multiplet formation was minimized by either method M2 or M3 (5%) while intermediate levels of multiplets were observed following preparation by methods M1 (24%) and M4 (20%). All other methods led to higher frequency of multiplet formation (46–73%) (Fig. 2c). Overall, methods M2 (WB pre-wash Fix/lyse) and M3 (WB Fix/lyse, no wash) provided data closest to the reference method M1 (WB diluted). Method M4 (WB formic acid lysis) does not induce degranulation; however, it results in significantly reduced CD62L levels and higher multiplet formation compared to M2, suggesting increased activation.

Importantly, methods M1-M4, i.e. the methods with low manipulation scores, result in low or moderate neutrophil activation, while methods with a high number of manipulation steps involving density gradients (M7-M10) exhibit the highest levels of neutrophil activation and degranulation (Table 1, 2b,c). A strong positive correlation was observed between the manipulation scores and levels of surface markers of neutrophil activation and degranulation (Fig. 2e), implicating manipulation steps as a major driver of neutrophil activation.

Next, we investigated whether characterization by different methods impacts the overall recovery of neutrophils, monocytes, and lymphocytes from whole blood samples. The highest cell recovery was observed following processing by methods M1-4 (Fig. 2d), likely due to the minimization of cell loss during washing and other processing steps. Neutrophil recovery was found to be most consistent between methods M3 and M4, neither of which requires washing during processing, thus preventing cell loss (Fig. 2d). Lymphocyte recovery was highest using methods M1, M3, M4, and M5, while monocyte recovery was highest using method M4 (Supplementary Fig. S1c). Overall, the results indicate that processing by a One-step Fixation and RBC Lysis buffer minimizes neutrophil activation, degranulation, and multiplet formation while preserving cell recovery.

### Removing unbound antibodies before RBC lysis and fixation reduces nonspecific binding

**Figure 3:**
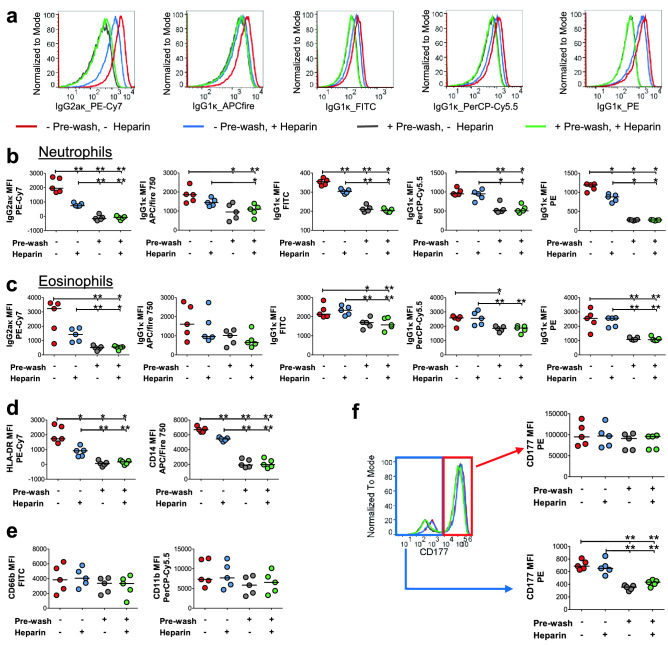
Removal of unbound antibodies prior to RBC lysis and fixation reduces nonspecific antibody binding. WB from healthy donors was collected in ACD tubes and staining was performed at 4 °C in the presence or absence of heparin with or without pre-washing to remove unbound antibodies before fixation and RBC lysis. (**a**) Representative histogram overlays of nonspecific antibody binding to neutrophils in the presence or absence of heparin with or without pre-washing step. (**b**, **c**) Nonspecific antibody binding to (**b**) neutrophils and (**c**) eosinophils. (**d**, **e**) Neutrophil surface levels of (**d**) low-level (HLA-DR and CD14) and (**e**) high-level (CD66b and CD11b) proteins. (**f**) Representative histogram overlay and quantification of neutrophil surface levels of CD177 on either CD177^high^ (top) or CD177^neg^ (bottom) neutrophils. Bars indicate medians; *p < 0.05, **p < 0.01; analyzed using Mann–Whitney test.

Most manufacturers’ protocols recommend the addition of Fixation/lysing buffers immediately following staining with antibodies still present in the mix. It was, therefore, critical to address the effect of fixation and RBC lysis in the presence of antibodies on nonspecific binding. Whole blood was stained for 30 min at 4 °C and unbound antibodies were either removed (referred to as “pre-wash”), as in method M2, or not removed as in method M3 prior to the addition of Fix/lyse buffer. All samples were washed before data acquisition by flow cytometry. Neutrophils that are subjected to Fix/lyse in the presence of fluorescently labeled antibodies, as recommended in the manufacturer’s protocol, displayed significantly increased binding of nonspecific antibodies used as isotype controls (Fig. 3a,b).

Neutrophils contain high quantities of cationic proteins including myeloperoxidase^34–37^ and defensins^38,39^ that may mediate nonspecific binding interactions with anionic regions of antibody constructs^40^. To test whether the observed nonspecific binding was caused by ionic interactions, whole blood was stained in the presence or absence of heparin, a negatively charged polymer with or without pre-washing as previously described^40^. Heparin treatment significantly reduced nonspecific binding of several antibody-fluorochrome conjugates to neutrophils although not to the same extent as pre-washing the antibodies (Fig. 3b). Heparin treatment exerted a less pronounced effect on nonspecific antibody binding to eosinophils, monocytes, or lymphocytes (Fig. 3c, Supplementary Fig. S2). Pre-washing the antibodies before treatment with Fix/lyse buffer significantly reduced nonspecific binding to neutrophils and other cell types, precluding any effect of heparin treatment (Fig. 3b,c, Supplementary Fig. S2). This is consistent with a contributing role of cationic proteins abundantly expressed in neutrophils in the facilitation of nonspecific antibody binding.

To determine how the nonspecific antibody binding impacts staining with antibodies specific to antigens with various levels of expression, the effect on the detection of low- (HLA-DR and CD14) versus moderate/high-level (CD66b and CD11b) neutrophil surface proteins with and without heparin incubation and pre-washing was evaluated. Heparin treatment significantly reduced the signal of surface antigens expressed at low levels (Fig. 3d). The moderate/high-level proteins displayed a trend of decreased staining after pre-washing; however, none of the conditions reached statistical significance (Fig. 3e). These data suggest that nonspecific binding has the most significant effect on antigens expressed at low levels.

To test this hypothesis directly, we utilized staining with CD177 (also named NB1, HNA-2a, or PRV-1), a surface protein present on a subset of neutrophils with genetically-regulated expression^41,42^, providing simultaneous readings for both CD177^neg^ and CD177^high^ populations (Fig. 3f). While the CD177^neg^ subset exhibited a significant reduction in signal following pre-washing similar to the nonspecific antibody stain, the CD177^high^ population did not differ in any of the conditions tested (Fig. 3f). Taken together, these data show that although a partial reduction of nonspecific antibody binding can be achieved with heparin treatment blocking interactions with cationic proteins, pre-washing unbound antibodies, as in method M2, is essential for accurate determination of the levels of neutrophil surface antigen, especially those expressed at low and moderate levels.

### The effect of staining temperature on neutrophil phenotype

**Figure 4:**
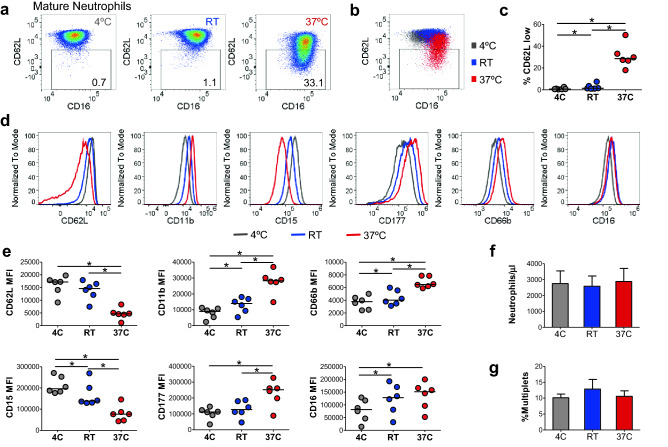
The effect of staining temperature on neutrophil phenotype. (**a**) Pseudocolor plots representing the staining of neutrophils from whole blood at the indicated temperatures. (**b**) Representative dot plot overlay of surface levels of CD16 and CD62L gated on neutrophils from WB incubated at the indicated temperatures. (**c**) Percentage of neutrophils with low surface levels of CD62L following staining at the indicated temperature. (**d**) Representative histogram overlays of neutrophil surface protein levels in fresh whole blood obtained from a healthy donor (HD) following staining at the indicated temperatures. CD177 histogram depicts CD177^high^ neutrophils only as gated in Fig. 3f. (**e**) Neutrophil surface protein levels in WB from HDs following staining at the indicated temperature. (**c**, **e**) Bars indicate medians *p < 0.05, **p < 0.01, analyzed by Wilcoxon matched-pairs signed rank test. (**f**) Number of neutrophils recovered per microliter of WB and (**g**) percent of neutrophils that formed multiplets in whole blood stained at the indicated temperatures. Bars represent mean/SEM *p < 0.05, **p < 0.01, analyzed using Wilcoxon matched-pairs signed rank test, n = 3.

To assess the effect of staining temperature on neutrophil phenotype, whole blood was stained for 30 min at either 4 °C, room temperature (RT), or 37 °C. Samples were then washed to remove unbound antibodies, treated with Fix/lyse buffer, washed, and cells were suspended in running buffer for analysis (method M2). On average, 1% and 3% of mature neutrophils displayed an activation-induced reduction of the surface level of CD62L following staining at 4 °C and RT, respectively, compared to 31% of neutrophils stained at 37 °C (Fig. 4a–c). The effect of staining temperature on neutrophil activation is further supported by significant increases of CD11b, CD66b, and CD16 and significant decreases of MFIs of CD15 and CD62L on total neutrophils at 37 °C (Fig. 4d,e). Despite the changes in neutrophil activation and degranulation, no changes were observed in the overall recovery of neutrophils or multiplet formation based on staining temperature (Fig. 4f,g). Together, these data suggest that staining at 4 °C minimizes neutrophil activation and degranulation and prevents alterations of neutrophil phenotype without impacting neutrophil recovery.

### The effects of sample processing delays and temperature on neutrophil phenotype

**Figure 5:**
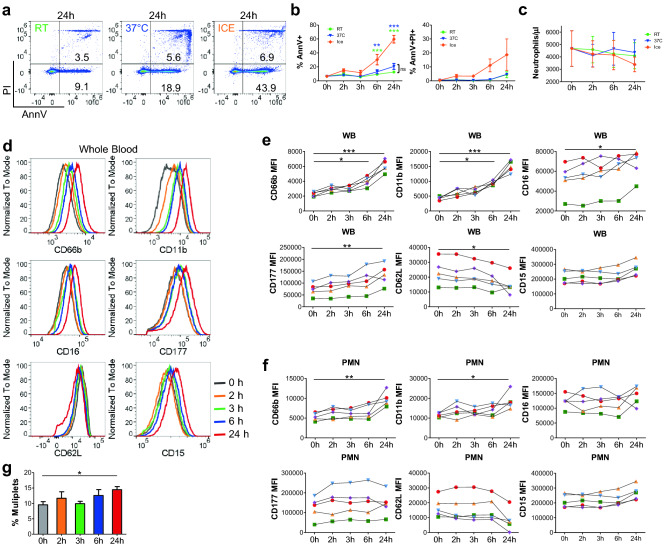
The impact of sample processing delays and temperature on neutrophil phenotype. (**a**) Representative plots of neutrophil apoptosis and viability following 24 h of WB incubation at the indicated temperatures. (**b**) Quantification of Annexin V^+^ (apoptotic, left) and Annexin V^+^/PI^+^ (late apoptotic/necrotic, right) neutrophils over time from WB incubated at the indicated temperatures. (**c**) Neutrophil recovery per microliter of WB over time following incubation at the indicated temperatures. (**b**, **c**) Results were compared using a two-way ANOVA, with Bonferroni post-tests *p < 0.05, **p < 0.01, ***p < 0.001, n = 3 per condition. (**d**) Representative histogram overlays of neutrophil surface protein levels assessed following RT incubation for the indicated amount of time following blood draw. (**e**, **g**) Surface levels of indicated proteins over time on neutrophils from (**e**) WB or (**f**) the PMN layer of a single Ficoll gradient. Each color represents measurements from the same sample over time, n = 5. (**g**) Comparison of the percentage of neutrophils forming multiplets in WB at the indicated time following sample collection, n = 6. (**e**–**g**) Results were compared using a Friedman test with Dunn’s multiple comparison post-hoc test *p < 0.05, **p < 0.01. All samples were collected in ACD tubes, incubated at RT, and stained at 4 °C unless otherwise indicated.

To compare the effect of delayed sample processing, the level of neutrophil apoptosis was determined immediately after blood draw and following incubation of whole blood on ice, at RT, or at 37 °C for 2, 3, 6, and 24 h. Neutrophil apoptosis detected by Annexin V (AnnV) staining was observed following 6- and 24-h incubation. The level of neutrophil apoptosis was significantly higher in blood samples incubated on ice (60%) compared to samples incubated at 37 °C (21%) or at RT (13%) (Fig. 5a,b). Consistent with previous studies investigating neutrophil apoptosis rates over time^43–45^, a subset of apoptotic neutrophils were positive for both Annexin V and propidium iodide (19% AnnV^+^PI^+^ on ice at 24 h), indicating that the neutrophils were in the early stage of apoptosis (Fig. 5a,b). This is consistent with the finding that neutrophil recovery does not significantly differ within 24 h among different incubation temperatures (Fig. 5c).

To assess the effect of post-draw incubation time on neutrophil phenotype, surface protein levels were assessed following RT incubation for 0, 2, 3, 6, or 24 h. Within 6 h of blood draw, neutrophil surface levels of CD66b and CD11b increased significantly, indicating degranulation (Fig. 5d,e). By 24 h after blood draw, elevated levels of markers of neutrophil activation including CD16 and CD177, decreased CD62L levels, and increased multiplet formation were observed (Fig. 5d,e,g). Interestingly, the changes were less pronounced in neutrophils collected from the PMN layer (Fig. 5f). Substantial activation during PMN preparation compared to WB demonstrated in Fig. 2 and evidenced here by the differences in surface marker levels (Fig. 5e,f) likely masks the increase in neutrophil activation over time. Overall, the collected data indicate that neutrophil phenotype can be accurately measured up to 3 h following blood draw but should not be delayed beyond that time without experiment-specific validation.

### Effects of anticoagulant type on neutrophil phenotype and recovery

**Figure 6:**
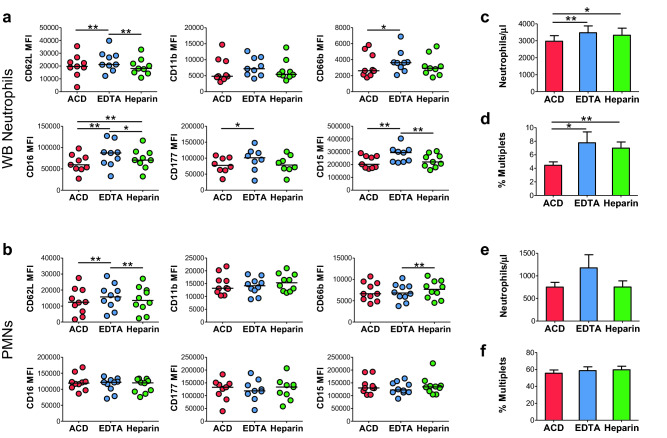
Effects of anticoagulant type on neutrophil phenotype and recovery. (**a**, **b**) Levels of surface proteins on neutrophils from (**a**) WB or (**b**) PMNs from HDs following collection from the same individual using ACD, EDTA, or heparin tubes. Bars indicate medians. (**c**) Neutrophil recovery per microliter or (**d**) percent multiplets formed by neutrophils from WB collected using the indicated tube types. Bars indicate means/SEM. (**e**) Neutrophil recovery per microliter or (**f**) percent multiplets formed by neutrophils from PMNs from WB collected using the indicated tube types. Bars represent means/SEM. For all panels, *p < 0.05, **p < 0.01 by Wilcoxon matched-pairs signed-rank tests, n = 9. All staining was performed at 4 °C.

Commonly used blood collection tubes utilize either anticoagulant citrate dextrose (ACD), ethylenediaminetetraacetic acid (EDTA), or heparin to prevent clotting. To address the inherent effect of anticoagulants on neutrophil phenotype, we analyzed neutrophils from each tube type drawn simultaneously from the same individual to minimize donor-specific variations. All samples were stained using method M2 at 4 °C. Neutrophils from whole blood collected in the presence of EDTA displayed significantly higher levels of CD62L, CD66b, CD16, CD177, and CD15 compared to blood collected in ACD or heparin tubes (Fig. 6a). The type of anticoagulant used for blood collection did not exert a discernable effect on neutrophil response to staining temperature as neutrophils collected in the presence of EDTA or heparin anticoagulants exhibited similar activation profiles following staining at different temperatures as did those collected in tubes utilizing ACD (Supplementary Fig. S3, Fig. 4d,e). Neutrophil recovery was significantly higher from blood collected in EDTA and heparin tubes than ACD tubes (Fig. 6c). Neutrophils from ACD tubes formed fewer multiplets compared to neutrophils from EDTA or heparin tubes (Fig. 6d).

Many studies utilize neutrophils collected from the PMN layer following preparation by either single or double discontinuous density gradient centrifugation methods that, as demonstrated in Fig. 1, result in a significantly activated cellular phenotype. We assessed the levels of activation and degranulation markers on neutrophils from each tube type as well as neutrophil recovery and multiplet formation following PMN layer isolation from a single Ficoll gradient (M8). While differences were observed in surface marker levels among different tube types, they were small and not consistent with findings in WB (Fig. 6a,b). No significant differences in neutrophil recovery or multiplet formation were detected (Fig. 6e,f). Similar changes in neutrophil phenotype were observed over time in neutrophils from WB or the PMN layer collected in ACD, EDTA, and heparin tubes, verifying that these changes are not specific to the anticoagulant used during collection (Fig. 5e,f, and Supplementary Fig. S4). Overall, this data suggests that the anticoagulant used for blood collection has a limited but discernible effect on the level of neutrophil activation and degranulation.

## Discussion

Neutrophils are essential innate immune cells well known for their rapid response to infection and injury. Prominent among the arsenal of antimicrobial functions of neutrophils is the rapid release of proteolytic enzymes and antimicrobial peptides through the mobilization of pre-formed intracellular granules. Neutrophils contain four major types of granules: azurophilic, specific, and gelatinous granules, and secretory vesicles. These granules differ in their cargo composition, membrane-bound proteins, and ease of release upon neutrophil activation, as recently reviewed by Yin and Heit^46^.

Progress in our understanding of neutrophil biology and heterogeneity in various disease states is hampered by a lack of consensus regarding optimal methods for the characterization of this sensitive and readily activated population. Here, we provide a comprehensive comparative analysis of neutrophil phenotypic changes measuring neutrophil activation and degranulation following preparation by eight commonly used methods of neutrophil characterization. Upon activation, CD62L is rapidly cleaved from the surface of neutrophils^29,30^ and granules are mobilized at varying rates^46^. Secretory vesicles are the most rapidly released granules following neutrophil activation, leading to the translocation of CD16 to the neutrophil surface, replacing and increasing the resting levels of CD16 that was cleaved from the surface upon activation^47,48^. The surface proteins CD66b and CD11b are commonly used as measures of neutrophil degranulation and are found in specific granules which are released less readily than secretory vesicles upon neutrophil activation^46,49^. Importantly, CD11b is found in secretory vesicles and gelatinous granules as well, allowing for the detection of earlier neutrophil degranulation^46,49^.

The data presented here indicate that the level of neutrophil activation and degranulation strongly correlates with the number of manipulation steps used during processing, suggesting that the manipulation itself may be largely responsible for the observed alterations of the phenotype. Neutrophils that undergo density gradient centrifugation demonstrate significant activation and degranulation while providing the lowest neutrophil recovery rate. We demonstrate that staining whole blood followed by a pre-wash step to remove unbound antibodies and treatment with One-step Fixation and RBC Lysis buffer (M2) minimizes neutrophil activation, degranulation, and multiplet formation during sample preparation. Importantly, the methods presented here are not intended to be a finalized guide to neutrophil preparation but rather a step towards higher quality standards of characterization of this critical cell population.

The data presented in this study demonstrate that several cell types are prone to nonspecific binding of antibodies during fixation and RBC lysis that significantly alters the detection levels of low-expression surface antigens. Heparin was previously shown to abrogate nonspecific binding of metal-conjugated antibodies, used for cytometry by time of flight (CyTOF) analysis, to eosinophils by disrupting interactions between anionic regions of the antibody constructs and cationic proteins found in eosinophils^40^. Interestingly, our data show that heparin treatment partially reduced nonspecific binding of some antibodies to neutrophils, suggesting that ionic interactions contribute to the process. The effect on nonspecific antibody binding to eosinophils was modest in our study, possibly due to the differences in electrochemical properties between fluorochrome versus metal conjugate-based antibodies. The level of nonspecific binding may be partially dependent on the conjugated fluorochrome since isotype antibodies of the same identity conjugated to different fluorochromes show varying levels of nonspecific binding. While nonspecific binding has little impact on surface proteins expressed at moderate to high levels, determination of low-level proteins is significantly affected in the presence of antibodies during fixation/RBC lysis. While pre-washing remaining antibodies prior to fixation provides an effective method to minimize the impact on the determination of levels of surface proteins, it is likely that prevention of nonspecific antibody binding will be more challenging in the process of intracellular staining making experiment-specific validation and proper controls essential.

Incubation at 4 °C limits the rate of cellular metabolism and other processes^50^. This is consistent with our finding that neutrophil activation and degranulation during staining were limited at 4 °C. However, long-term blood storage on ice induced high levels of apoptosis and decreased overall cell viability. Based on these results, when delayed processing cannot be avoided, for example during shipment, the samples should be kept at room temperature rather than on ice or at 4 °C.

Although previous studies have shown that the functional capacity of neutrophils diminishes over time^23,26^, the effect of delayed processing on neutrophil phenotype has not been fully characterized. The data reported here demonstrate that when whole blood is incubated at RT, i.e. in conditions with the lowest rate of apoptosis, neutrophil activation and degranulation markers are significantly elevated by 6 hours, suggesting that, for most accurate results, neutrophil phenotype should be assessed within 3 hours of blood draw.

While previous reports show that anticoagulants impact the functional capacity of neutrophils^27,51,52^, the specific impact of anticoagulants on neutrophil phenotype has not been systematically characterized. Here we compared three common anticoagulants, ACD, EDTA, and heparin. Although our findings show that the levels of some surface proteins were altered in the EDTA samples compared to heparin or ACD, the observed differences were relatively minor and may reflect differences in antibody binding kinetics rather than neutrophil activation. Based on these results, it is likely that most anticoagulants can reliably be used for phenotyping; however, the use of multiple anticoagulants within a specific study should be avoided.

Limitations of this study include the number of participants and antigens tested. The sensitivity to time and temperature-associated changes in phenotype varied among different antigens, highlighting the importance of experiment-specific validation. Additionally, not all neutrophil characterization and isolation methods were directly tested here. While the amount of activation and degranulation induced by each may be predicted by the manipulation steps required, additional validation will be required in future studies. Although the presented study utilized samples from healthy donors, similar results were obtained using samples from HIV-1 and SARS-CoV-2-infected individuals (data not shown). Future studies will be required to elucidate the specific effect of incubation time and preparation-induced neutrophil activation and degranulation on other important functional readouts including phagocytosis, chemotaxis, and production of specific mediators.

Overall, our data highlight the importance of rapid sample processing with minimal manipulation and removal of remaining unbound antibodies prior to fixation/lysis (method M2) for reliable preservation of neutrophil phenotype. Specific disease settings may exert additional effects on the rate and magnitude of phenotypic changes of neutrophils under various experimental conditions, highlighting the importance of careful validation of experimental conditions and analytical approaches within specific studies.

## Methods

### Sample collection from human subjects

All methods were performed in accordance with the relevant guidelines and regulations. The study protocol was approved by the Institutional Review Board of the University of Alabama at Birmingham (IRB protocol # 141218001). Peripheral blood was collected from healthy donors following informed consent. Blood was collected by certified phlebotomists in collection tubes that utilize acid citrate dextrose (ACD, Fisher Scientific, Waltham, MA, USA), ethylenediaminetetraacetic acid (EDTA, Fisher Scientific), or heparin (Fisher Scientific) as indicated. Unless otherwise specified, sample processing began within 1 h of blood draw.

### WB staining and dilution (M1)

Fifty microliters of fresh whole blood was incubated with 50 µl of antibody mix (all antibodies in Dulbecco’s Phosphate Buffered Saline (DPBS, Corning, Manassas, VA, USA) with 10% human serum type AB (HS, FisherBioReagents, Waltham, MA, USA)) at 4 °C for 30 min. Following incubation and gentle mixing, a 4 µl aliquot of the suspension (i.e. 2 µl WB and 2 µl antibody mixture) was transferred to 4 ml of DPBS for a 1:2000 final dilution of whole blood. The samples were analyzed immediately by multi-parametric flow cytometry with acoustic focusing technology (Attune NxT, ThermoFisher Scientific, Waltham, MA, USA).

### WB stain, pre-wash, fix/lyse (M2)

Fifty microliters of fresh whole blood was incubated with 50 µl of antibody mix (all antibodies in DPBS with 10% HS) at 4 °C for 30 min. After staining, 4 ml of cold DPBS with 1 mM EDTA (Corning) was added to the cell suspension, gently mixed with a 1 ml pipette and centrifuged at 200×*g* for 5 min to wash away unbound antibodies. Supernatant was aspirated and the cells were gently resuspended in 1 ml of RT 1-step Fix/lyse buffer (Invitrogen, Waltham, MA, USA). The suspension was incubated for 15 min at room temperature to lyse RBCs and simultaneously fix other cells. Following RBC lysis, 1 ml cold (4ºC) DPBS with 1 mM EDTA was added to the samples, the cells were gently mixed and centrifuged at 200×*g* for 5 min. Supernatant was aspirated and samples were resuspended in 250 µl of cold (4 °C) Wash Buffer [DPBS with 2% FBS (Atlanta Biologics, Flowery Branch, GA, USA)] and 250 µl of cold (4 °C) Intracellular (IC) Fixation buffer (Invitrogen) was added on top. Samples were gently mixed and stored at 4 °C until analysis (within 24 h) by multi-parametric flow cytometry.

### WB stain, fix/lyse (M3)

Fifty microliters of whole blood was incubated with 50 µl of antibody mix at 4 °C for 30 min. After staining, 1 ml of RT 1-step Fix/lyse buffer was added (Invitrogen). RBCs were lysed and other cells fixed for 15 min at room temperature. Following RBC lysis, 1 ml cold (4 °C) DPBS with 1 mM EDTA was added to the samples followed by centrifugation at 200×*g* for 5 min. Supernatant was aspirated and samples were re-suspended in 250 µl of cold (4 °C) Wash buffer, 250 µl of cold (4 °C) Intracellular (IC) Fixation buffer (Invitrogen) was added, gently mixed, and samples were stored at 4 °C until analysis (within 24 h) by multi-parametric flow cytometry.

In the absolute count samples, following staining and Fix/lyse incubation, 50 µl of well-mixed CountBright™ Absolute Counting Beads (RT) (ThermoFisher Scientific) were added according to the manufacturer’s protocol and samples were vortexed gently and stored at 4 °C until analysis with no additional processing (for absolute count calculations, see “Cell recovery and multiplet calculations” section).

### WB formic acid lysis (M4)

Fifty microliters of whole blood was incubated with 50 µl of antibody mix at 4 °C for 30 min. The sample was placed on a vortex and 600 µl formic acid lysis buffer (600 µl 98% formic acid (Millipore Sigma, Darmstadt, Germany) in 500 ml of distilled water) was added, the sample was gently vortexed for 15 s, 200 µl of the Stop solution (6 g Na_2_CO_3_, 14.5 g NaCl, 31.3 g Na_2_SO_4_, 1 g NaN_3_ in 1 l final volume of distilled water) was immediately added, the sample was gently vortexed for 15 s, 128.5 µl of 16% paraformaldehyde (PFA) was immediately added (2% PFA final) and the sample was gently vortexed for an additional 15 s. Samples were stored at 4 °C until analysis (less than 24 h) by multi-parametric flow cytometry.

### WB sedimentation (M5)

Four milliliters of whole blood and 2 ml of RT Sedimentation Buffer (Miltenyi, Bergisch Gladbach, Germany) were combined in a 15 ml conical tube. The tube was rotated gently for 5 min before the cap was removed and the sample left to stand (RT) for 15 min to allow for RBC sedimentation. The supernatant was collected into a new 15 ml tube and RBC depletion beads (RT, 20 µl per original ml of WB) (Stem Cell Technologies, Vancouver, Canada) were added. The cell suspension was rotated gently for 5 min and placed in a magnet for 10 min with cap off to separate the magnetic beads and remaining RBCs from the cell suspension. The supernatant was transferred to a new tube and centrifuged for 10 min at 300×*g* to pellet the cells. The supernatant was aspirated and cells were suspended in cold (4 °C) Staining buffer (DPBS with 10% HS) for blocking at 4 °C for 30 min. During blocking, the cells were counted and diluted so that 1 million cells were aliquoted per 50 µl for each stain. Fifty microliters of antibody mix was added to each 50 µl cell suspension aliquot, cells were stained at 4 °C for 30 min, washed with 2 ml cold (4 °C) Wash buffer and centrifuged for 5 min at 200×*g*. The supernatant was aspirated and the cells were suspended in 250 µl of Wash buffer, 250 µl of cold (4 °C) IC fixation buffer was added, and samples were gently mixed and stored at 4 °C until analysis (within 24 h) by multi-parametric flow cytometry.

### WB isotonic lysis (M6)

Ice-cold isotonic NH_4_Cl solution (isotonic RBC lysis buffer: 155 mM NH_4_Cl, 10 mM KHCO_3_, and 0.1 mM EDTA), was prepared weekly and vacuum filtered as previously reported by Kleijn, et al.^53^. Briefly, 2.5 ml of whole blood was diluted 1:1 with cold (4 °C) 2.5 mM EDTA in DPBS in a 15 ml conical tube and centrifuged at 300×*g* for 10 min. The supernatant was aspirated without disturbing the buffy coat or RBC pellet, the pellet was disrupted by gentle pipetting, and the tube was filled with 14 ml of cold (4 °C) isotonic lysis buffer. The tube was rotated for 5 min to lyse RBCs and centrifuged at 300×*g* for 10 min. The supernatant was discarded and the cells were washed with 10 ml cold (4 °C) Wash Buffer, centrifuged, and suspended in cold (4ºC) Staining buffer for blocking for 30 min at 4 °C. Cells were counted during blocking and 1 million cells in 50 µl were utilized for each stain. Cells were stained with 50 µl antibody mix for 30 min at 4 °C, washed with 2 ml cold (4 °C) Wash buffer, centrifuged (200×*g* for 5 min), suspended in 250 µl of cold (4 °C) Wash buffer, and 250 µl of cold (4 °C) IC fixation buffer was added and gently mixed. Samples were stored at 4 °C until analysis by multi-parametric flow cytometry (within 24 h).

### Single gradient (M7 and M8)

Whole blood was diluted 1:1 with RT DPBS and 8 ml of the suspension was layered onto a 4 ml discontinuous density gradient (RT, 1.077 g/ml Ficoll-Paque, Fisher Scientific). The gradient was centrifuged for 30 min at 400×*g* with the brake off. For PBMC isolation (M7): the PBMC layer located at the Ficoll-plasma interface was collected into 10 ml DPBS and washed with 10 ml DPBS twice (300×*g* for 10 min, brake on) and then suspended in cold (4 °C) Staining buffer. For PMN isolation (M8): the remaining Ficoll was removed, the RBC/PMN pellet was disrupted by gentle pipetting, and RBCs were lysed with cold (4 °C) Isotonic Lysis Buffer as detailed above. Both PMNs (M8) and PBMCs (M7) were blocked in cold (4 °C) Staining buffer for 30 min at 4 °C during which time the cells were counted and diluted to a concentration of 1 million cells per 50 µl for each stain. Cells were stained with 50 µl antibody mix for 30 min at 4 °C, washed with 2 ml cold (4 °C) Wash buffer, centrifuged (5 min, 200×*g*), and suspended in 250 µl cold (4 °C) Wash buffer and 250 µl cold (4 °C) IC Fixation buffer was added and gently mixed. Samples were stored at 4 °C until analysis (within 24 h) by multi-parametric flow cytometry.

### Double gradient (M9 and M10)

For the isolation of PBMCs and PMNs from a double discontinuous density gradient, 3 ml of RT 1.077 g/ml HISTOPAQUE-1077 (Sigma-Aldrich, St. Louis, MO, USA) was layered on top of 3 ml of RT 1.119 g/ml HISTOPAQUE-1119 (Sigma-Aldrich) in a 15 ml conical tube. Next, 6 ml WB was layered onto the top and the gradient was centrifuged for 30 min at 700×*g* with the brake off. The PBMC (at the Histopaque- plasma interface) and PMN (at the interface of the two Histopaque gradients) layers were each collected into 10 ml of DPBS and centrifuged (10 min, 300×*g*, brake on). PBMCs (M9) were washed twice while the PMNs (M10) underwent the isotonic lysis protocol outlined above to remove contaminating RBCs. Both samples were blocked in cold (4 °C) Staining buffer for 30 min and 1 million cells were aliquoted in 50 µl per stain. Cells were stained with 50 µl of antibody mix for 30 min at 4 °C. The samples were then washed with 2 ml cold (4 °C) Wash buffer, centrifuged (5 min, 200×*g*), resuspended in 250 µl of cold (4 °C) Wash buffer, 250 µl of cold (4 °C) IC Fixation buffer was added and the cells were gently mixed. Samples were stored at 4 °C until analysis (within 24 h) by multi-parametric flow cytometry.

### Heparin blocking

Where indicated, whole blood was incubated with 400 U/ml heparin (Sigma-Aldrich, CAS# H3393) for 20 min at 4 °C before staining and processing as indicated above.

### Annexin V and propidium iodide staining

An RBC depletion kit (Stem Cell Technologies) was adapted for use with 100 µl of WB. First, 3 ml of RT DPBS with 2.5 mM EDTA was added to 100 µl WB and 20 µl of depletion beads were added. The samples were gently mixed, incubated for 5 min, placed in a magnet for 5 min (no cap), and the supernatant collected into a fresh tube. Another 20 µl of depletion beads were added, incubated for 5 min, and then placed in a magnet for 5 min (no cap). The supernatant was transferred to a new tube and placed in a magnet for 5 min (no cap). The supernatant was transferred to a new tube and centrifuged (5 min, 200×*g*); the resulting supernatant was aspirated. Apoptosis was measured according to the manufacturer’s protocol using the Annexin V Apoptosis Detection Kit with PI (Biolegend, San Diego, CA, USA). Briefly, cells were suspended in 500 µl of RT Annexin V binding buffer and five microliters each of Annexin V antibody and propidium iodide (PI) were added to the tube. Cells were stained at room temperature for 15 min before an additional 500 µl of Annexin V binding buffer was added and the samples were analyzed immediately by flow cytometry.

### Flow cytometry

Cells were stained as indicated with the following combinations of antibodies (additional information can be found in Supplemental Table S1): (1) for cell activation, CD3-BV605, CD11b-PerCP-Cy5.5, CD14-APC/Fire^TM^750, CD15-eFluor450, CD16-APC, CD19-BV605, CD62L-BV711, CD66b-FITC, CD177-PE, CD193(CCR3)-BV510, HLA-DR-PE-Cy7, (2) for cell recovery calculations, CD3-FITC, CD4-PE, CD8a-PerCP-Cy5.5, CD14-APC/Fire^TM^750, CD15-eFluor450, CD16-APC, and CD19-BV605, and for nonspecific background controls, CD3-BV605, IgG1κ-PerCP-Cy5.5, IgG1κ-APC/Fire^TM^750, CD15-eFluor450, CD16-APC, CD19-BV605, CD62L-BV711, IgG1κ-FITC, IgG1κ-PE, CD193(CCR3)-BV510, and IgG2aκ- PE-Cy7. Following processing, samples were acquired using an Attune NxT flow cytometer (Thermo Fisher Scientific) and data (FCS) were analyzed using FlowJo 10.6.1 (Becton, Dickinson and Company, Ashland, OR, USA).

### Cell recovery and multiplet calculations

Each cell type’s recovery following processing by methods M1 and M2 were calculated based on the cells acquired per volume by flow cytometry as demonstrated in Eq. (1) for neutrophils where the dilution factor is calculated as the final volume analyzed by flow cytometry divided by the original volume of whole blood.

## Equation (1) for M1 and M2:

1 $$Neutrophils\ Recovered\ per \mu l\ WB=\left(\frac{neutrophil\ count\ by\ flow\ cytometry}{run\ volume, \mu l}\right) \times dilution\ factor$$

Cell recovery for M3 and M4 was determined using CountBright Absolute Counting Beads (Invitrogen) in whole blood per the manufacturer’s protocol since washing was not required. An example for calculating neutrophil recovery is shown in Eq. (2) below. The number of neutrophils and beads recovered are based on flow cytometry data while beads expected is based on the number of beads expected to be present in the 50 µl of bead suspension added to each sample.

## Equation (2) for M3 and M4:

2 $$Neutrophils\ Recovered\ per \mu l\ WB=\frac{\left(\#neutrophils\right)*\left(\frac{beads\ expected}{beads\ recovered}\right)}{50}$$

For methods M5-M10, cell recovery was calculated as demonstrated for neutrophils in Eq. (3). The total leukocytes recovered is based on cell counts after processing and the percentage of the cell type of interest out of total leukocytes is determined by flow cytometry. Gradient recovery is calculated by Eq. (3) for each the PBMC and PMN layers which are then added to give a total neutrophil recovery from whole blood.

## Equation (3) for M5–M10:

3 $$Neutrohils\ Recovered\ per \mu l\ WB =\left(\frac{total\ leukocytes\ recovered}{starting\ WB\ volume, \mu l}\right)*\%neutrophils\ of\ leukocytes$$

To determine how many neutrophils form multiplets during processing, neutrophils were identified as CD15 + cells and percent multiplets were determined based on single-cell gating utilizing forward scatter area and height as depicted in Supplemental Fig. S1b.

### Statistical analyses

Statistical significance was analyzed in GraphPad Prism 5.04 software using Mann–Whitney tests, linear regression analyses, Wilcoxon matched pairs signed-rank test, or Two-way ANOVAs with Bonferroni multiple comparisons post-tests as indicated in the text and figure legends.

## Supplementary Information

Supplementary Information.

## Data Availability

Datasets utilized in this study will be provided by the corresponding author upon reasonable request.
