# Supplementary material for: Optimization of methods for the accurate characterization of whole blood neutrophils
Source: Sci Rep. 2022 Mar 7;12:3667. doi: 10.1038/s41598-022-07455-2 (PMC8901620; doi:10.1038/s41598-022-07455-2)
Supplement: Supplementary file 1 — Supplementary Information. [file 41598_2022_7455_MOESM1_ESM.pptx]

## Slide 1
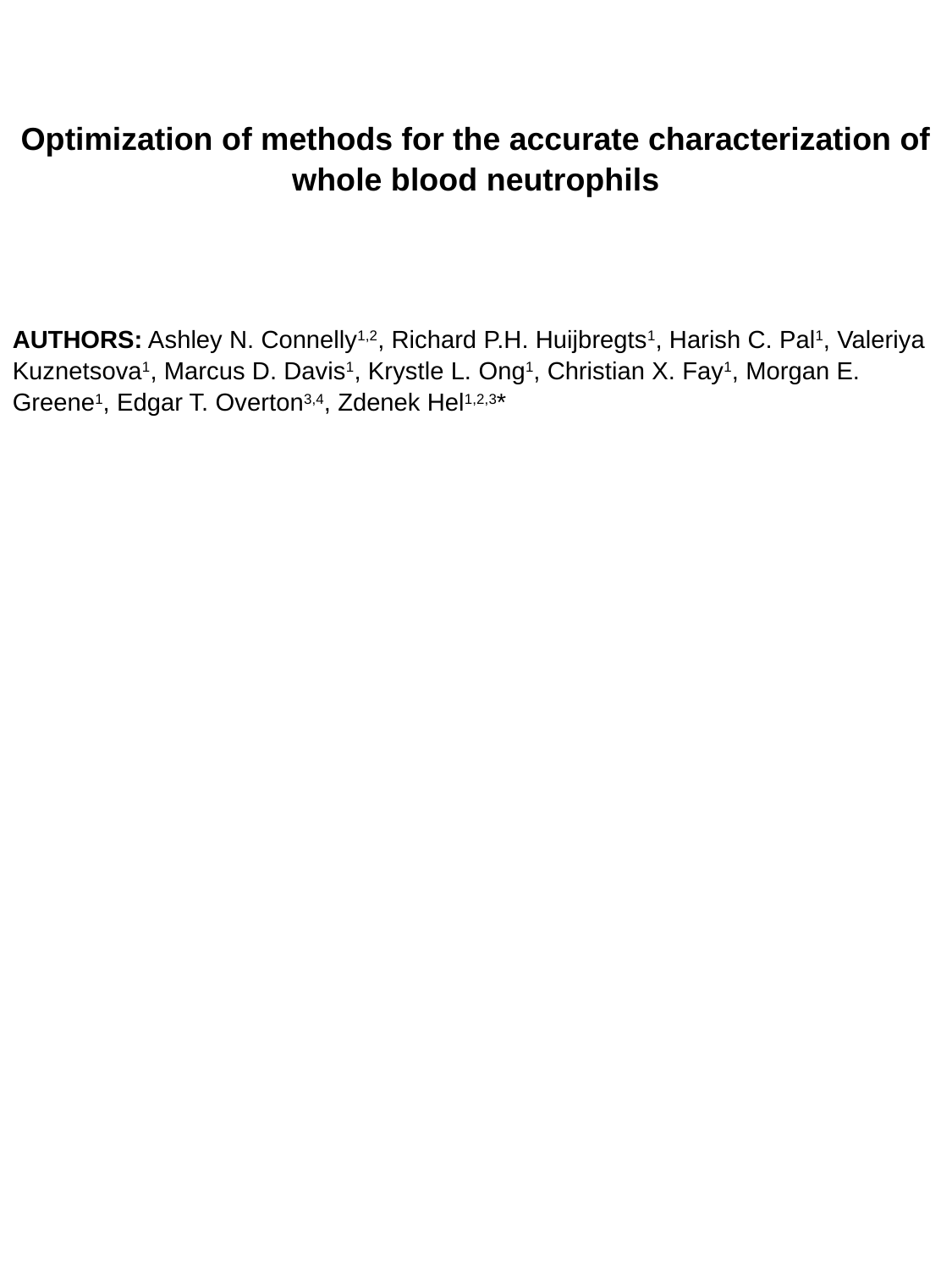

Optimization of methods for the accurate characterization of whole blood neutrophils
AUTHORS: Ashley N. Connelly1,2, Richard P.H. Huijbregts1, Harish C. Pal1, Valeriya Kuznetsova1, Marcus D. Davis1, Krystle L. Ong1, Christian X. Fay1, Morgan E. Greene1, Edgar T. Overton3,4, Zdenek Hel1,2,3*

## Slide 2
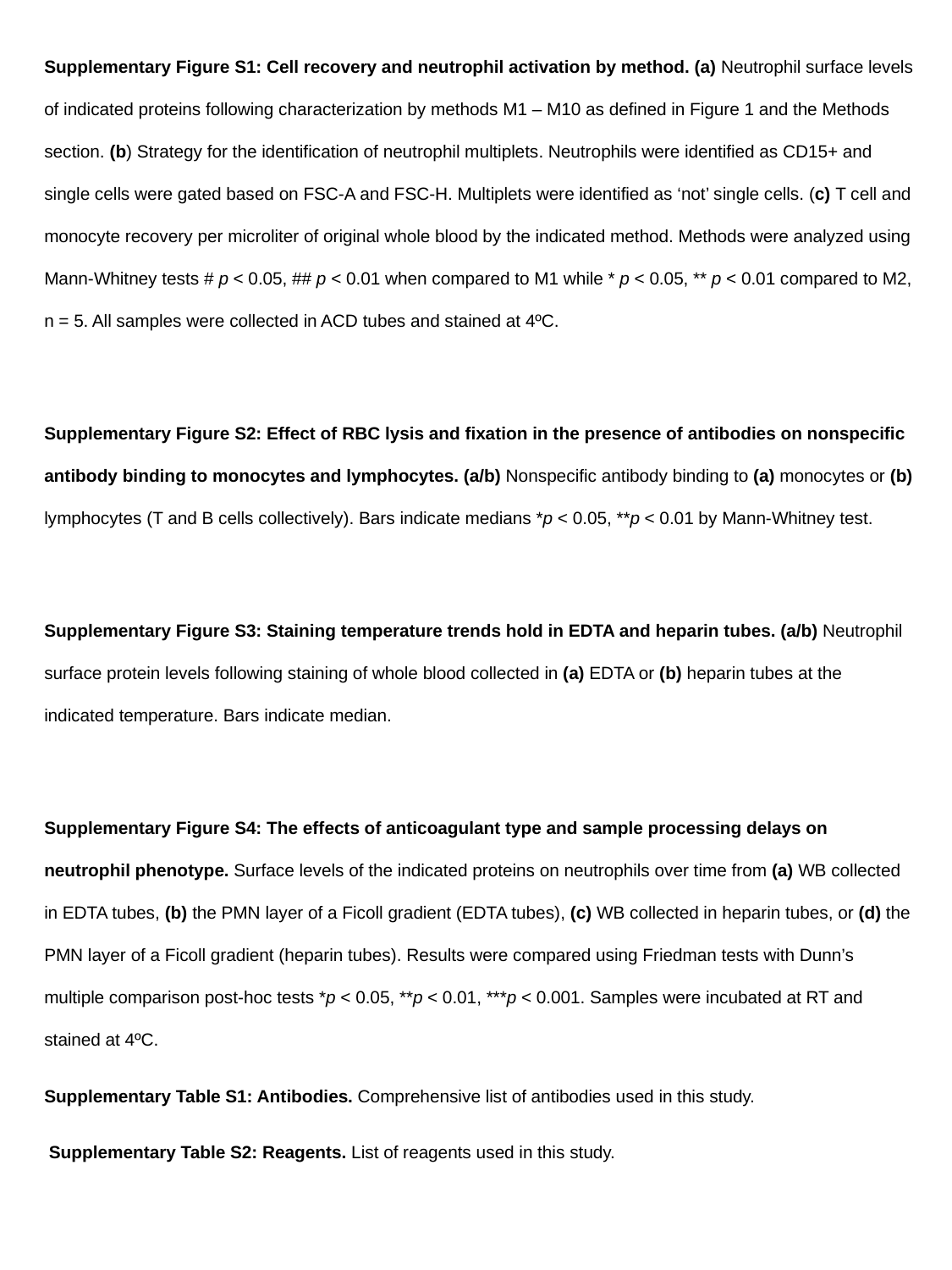

Supplementary Figure S1: Cell recovery and neutrophil activation by method. (a) Neutrophil surface levels of indicated proteins following characterization by methods M1 – M10 as defined in Figure 1 and the Methods section. (b) Strategy for the identification of neutrophil multiplets. Neutrophils were identified as CD15+ and single cells were gated based on FSC-A and FSC-H. Multiplets were identified as ‘not’ single cells. (c) T cell and monocyte recovery per microliter of original whole blood by the indicated method. Methods were analyzed using Mann-Whitney tests # p < 0.05, ## p < 0.01 when compared to M1 while * p < 0.05, ** p < 0.01 compared to M2, n = 5. All samples were collected in ACD tubes and stained at 4ºC.
Supplementary Figure S2: Effect of RBC lysis and fixation in the presence of antibodies on nonspecific antibody binding to monocytes and lymphocytes. (a/b) Nonspecific antibody binding to (a) monocytes or (b) lymphocytes (T and B cells collectively). Bars indicate medians *p < 0.05, **p < 0.01 by Mann-Whitney test.
Supplementary Figure S3: Staining temperature trends hold in EDTA and heparin tubes. (a/b) Neutrophil surface protein levels following staining of whole blood collected in (a) EDTA or (b) heparin tubes at the indicated temperature. Bars indicate median.
Supplementary Figure S4: The effects of anticoagulant type and sample processing delays on neutrophil phenotype. Surface levels of the indicated proteins on neutrophils over time from (a) WB collected in EDTA tubes, (b) the PMN layer of a Ficoll gradient (EDTA tubes), (c) WB collected in heparin tubes, or (d) the PMN layer of a Ficoll gradient (heparin tubes). Results were compared using Friedman tests with Dunn’s multiple comparison post-hoc tests *p < 0.05, **p < 0.01, ***p < 0.001. Samples were incubated at RT and stained at 4ºC.
Supplementary Table S1: Antibodies. Comprehensive list of antibodies used in this study.
 Supplementary Table S2: Reagents. List of reagents used in this study.

## Slide 3
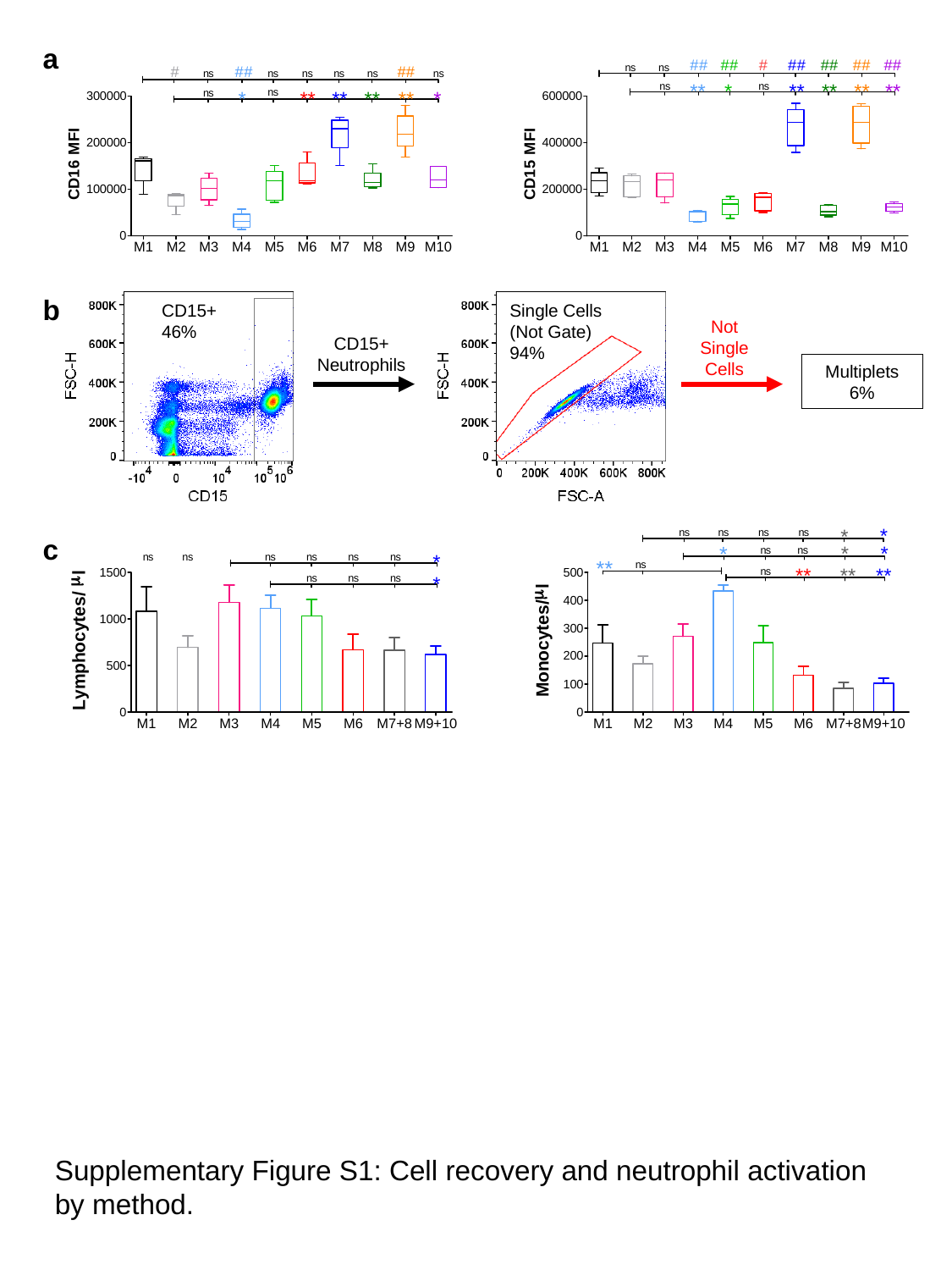

a
Single Cells
(Not Gate)
94%
CD15+
46%
b
Not Single Cells
CD15+ Neutrophils
Multiplets
6%
c
Supplementary Figure S1: Cell recovery and neutrophil activation by method.

## Slide 4
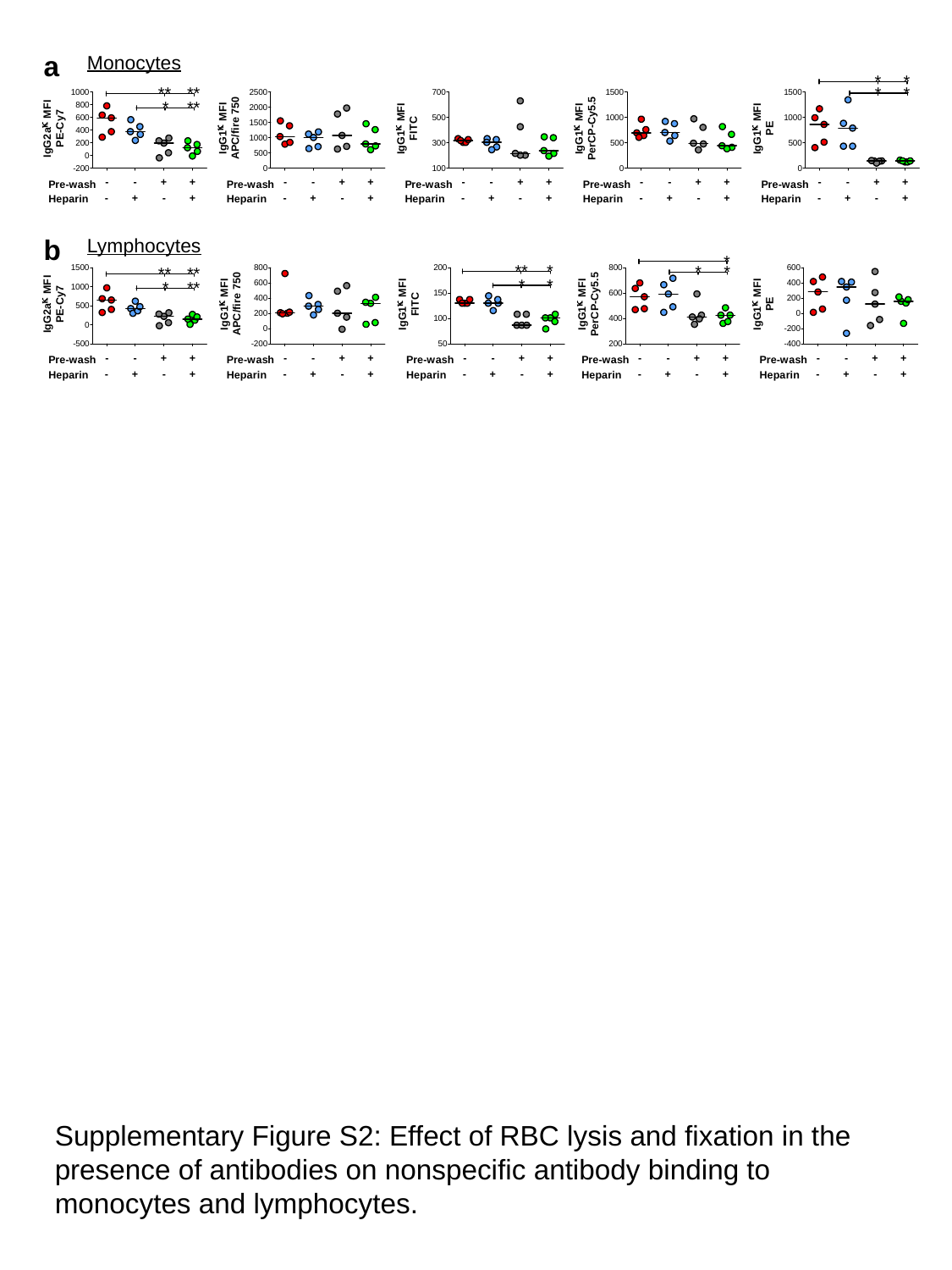

a
Monocytes
b
Lymphocytes
Supplementary Figure S2: Effect of RBC lysis and fixation in the presence of antibodies on nonspecific antibody binding to monocytes and lymphocytes.

## Slide 5
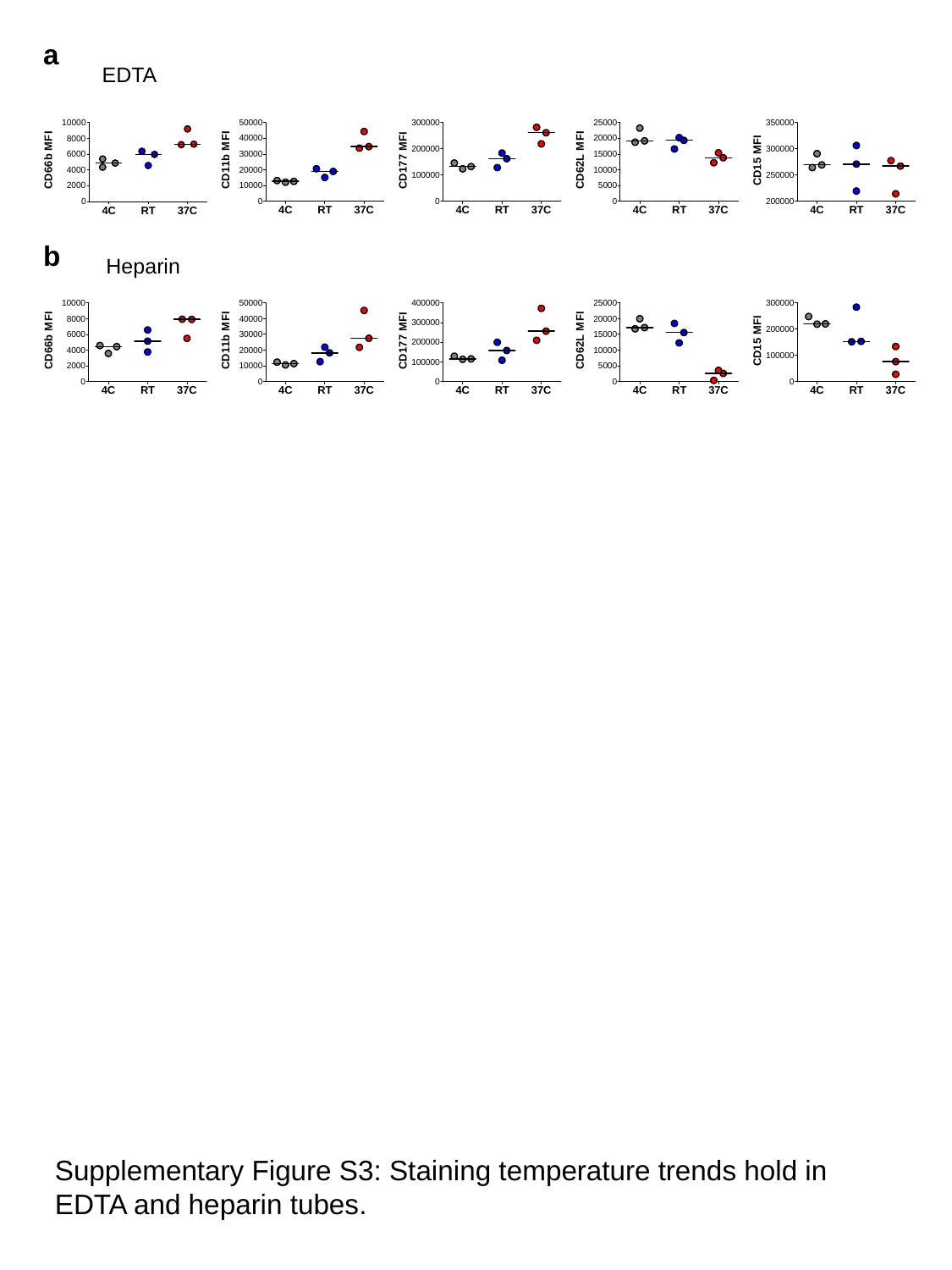

a
EDTA
b
Heparin
Supplementary Figure S3: Staining temperature trends hold in EDTA and heparin tubes.

## Slide 6
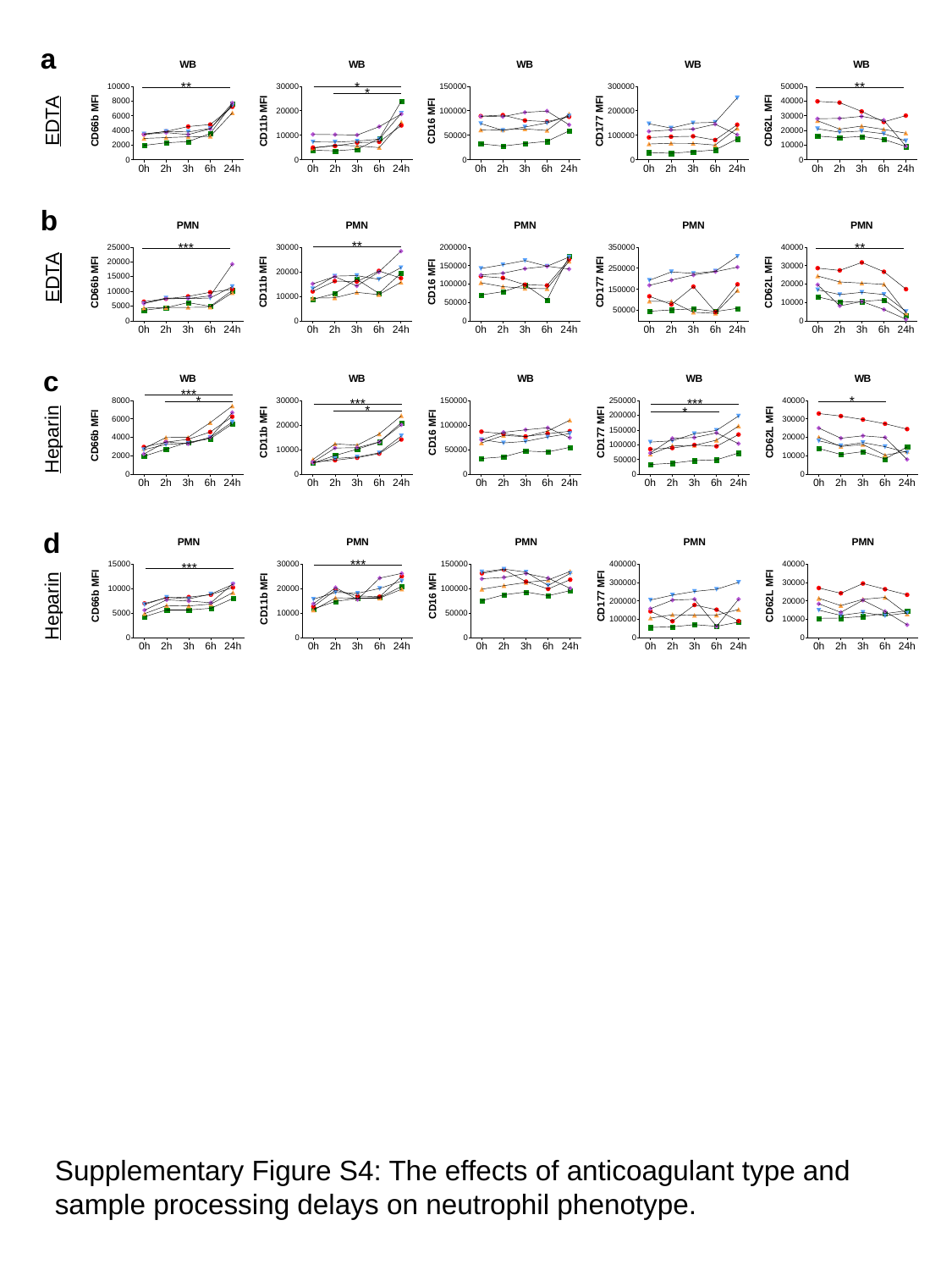

a
EDTA
b
EDTA
c
Heparin
d
Heparin
Supplementary Figure S4: The effects of anticoagulant type and sample processing delays on neutrophil phenotype.

## Slide 7
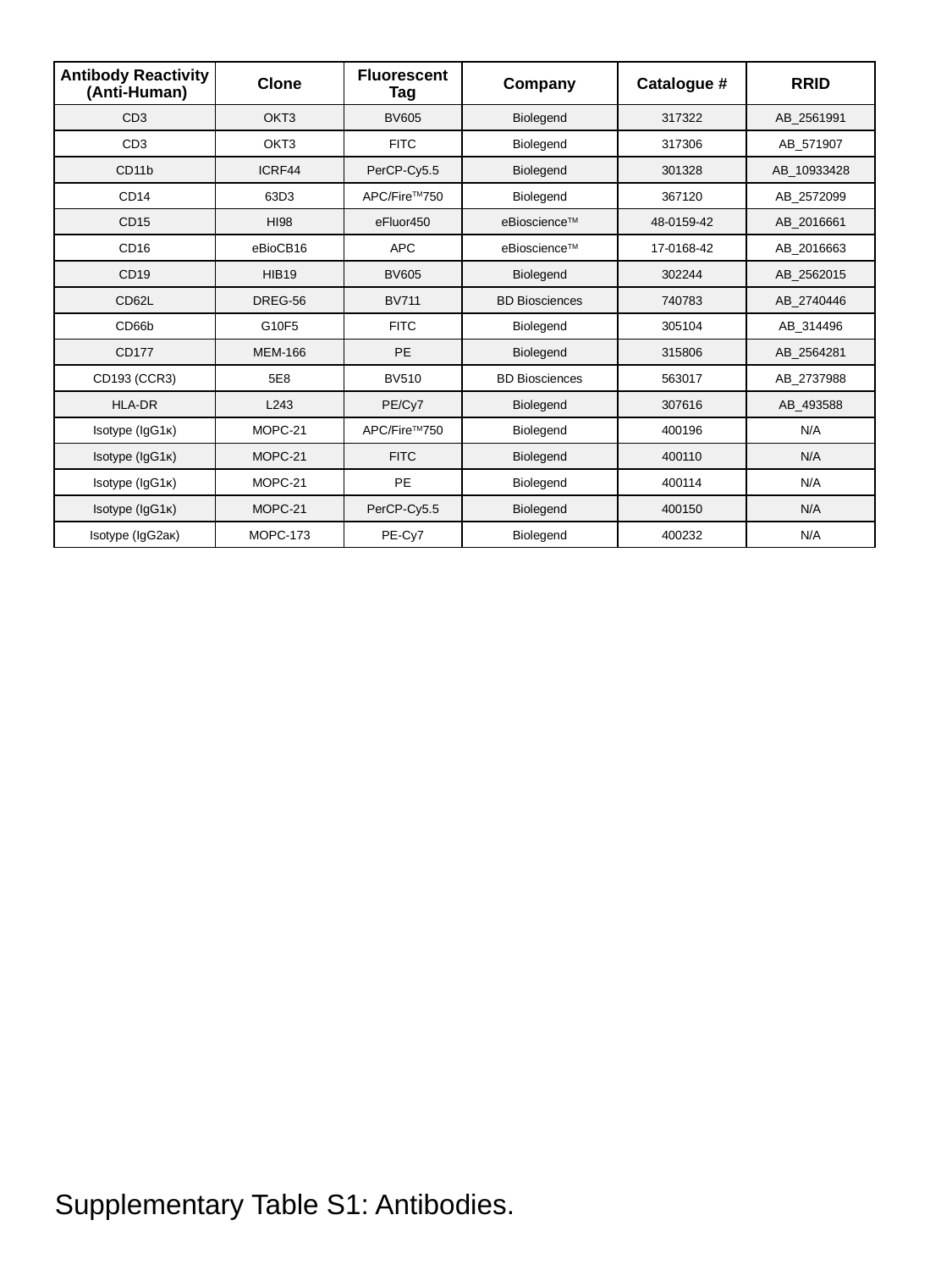

| Antibody Reactivity (Anti-Human) | Clone | Fluorescent Tag | Company | Catalogue # | RRID |
| --- | --- | --- | --- | --- | --- |
| CD3 | OKT3 | BV605 | Biolegend | 317322 | AB\_2561991 |
| CD3 | OKT3 | FITC | Biolegend | 317306 | AB\_571907 |
| CD11b | ICRF44 | PerCP-Cy5.5 | Biolegend | 301328 | AB\_10933428 |
| CD14 | 63D3 | APC/FireTM750 | Biolegend | 367120 | AB\_2572099 |
| CD15 | HI98 | eFluor450 | eBioscienceTM | 48-0159-42 | AB\_2016661 |
| CD16 | eBioCB16 | APC | eBioscienceTM | 17-0168-42 | AB\_2016663 |
| CD19 | HIB19 | BV605 | Biolegend | 302244 | AB\_2562015 |
| CD62L | DREG-56 | BV711 | BD Biosciences | 740783 | AB\_2740446 |
| CD66b | G10F5 | FITC | Biolegend | 305104 | AB\_314496 |
| CD177 | MEM-166 | PE | Biolegend | 315806 | AB\_2564281 |
| CD193 (CCR3) | 5E8 | BV510 | BD Biosciences | 563017 | AB\_2737988 |
| HLA-DR | L243 | PE/Cy7 | Biolegend | 307616 | AB\_493588 |
| Isotype (IgG1κ) | MOPC-21 | APC/FireTM750 | Biolegend | 400196 | N/A |
| Isotype (IgG1κ) | MOPC-21 | FITC | Biolegend | 400110 | N/A |
| Isotype (IgG1κ) | MOPC-21 | PE | Biolegend | 400114 | N/A |
| Isotype (IgG1κ) | MOPC-21 | PerCP-Cy5.5 | Biolegend | 400150 | N/A |
| Isotype (IgG2aκ) | MOPC-173 | PE-Cy7 | Biolegend | 400232 | N/A |
Supplementary Table S1: Antibodies.

## Slide 8
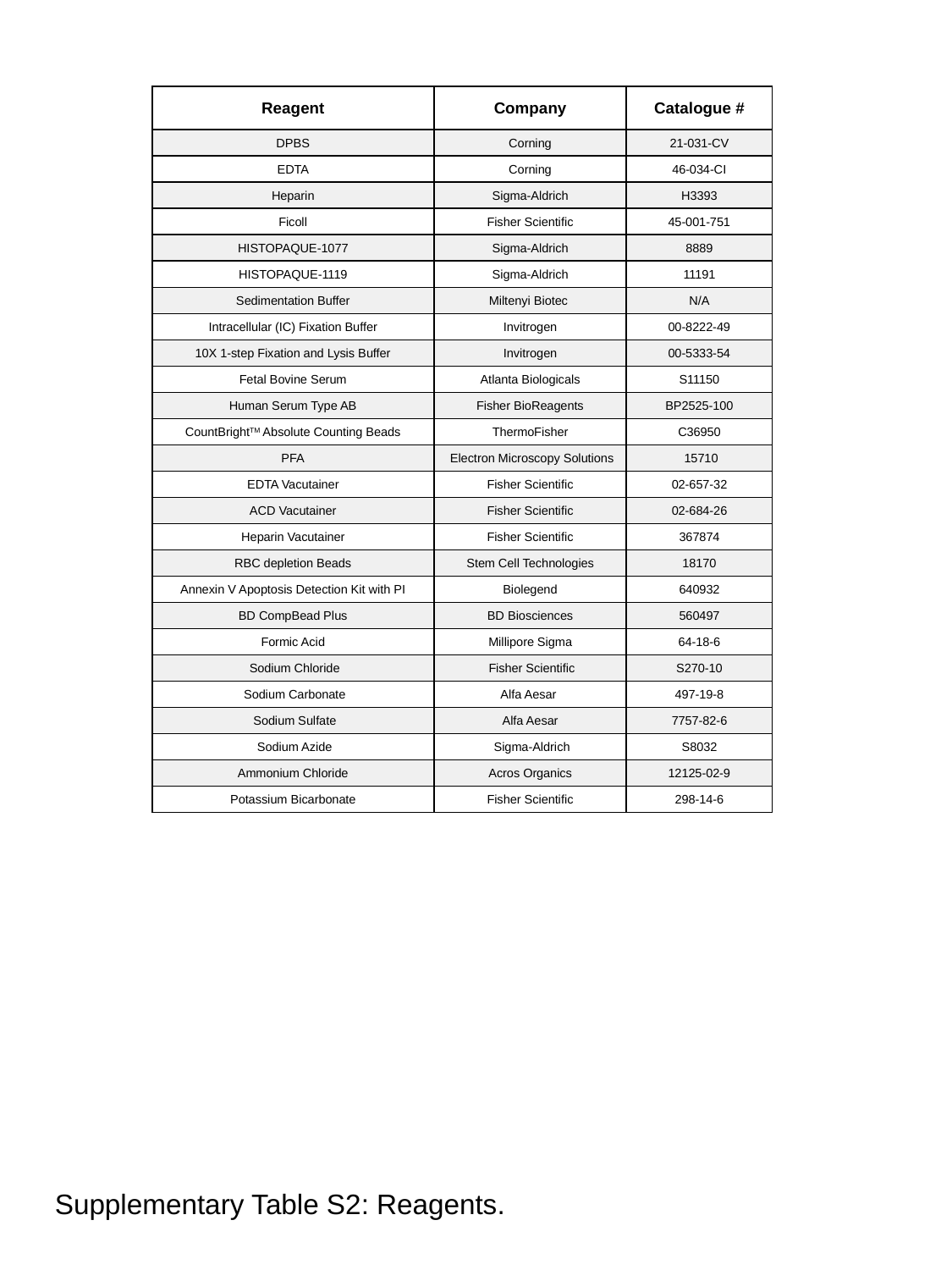

| Reagent | Company | Catalogue # |
| --- | --- | --- |
| DPBS | Corning | 21-031-CV |
| EDTA | Corning | 46-034-CI |
| Heparin | Sigma-Aldrich | H3393 |
| Ficoll | Fisher Scientific | 45-001-751 |
| HISTOPAQUE-1077 | Sigma-Aldrich | 8889 |
| HISTOPAQUE-1119 | Sigma-Aldrich | 11191 |
| Sedimentation Buffer | Miltenyi Biotec | N/A |
| Intracellular (IC) Fixation Buffer | Invitrogen | 00-8222-49 |
| 10X 1-step Fixation and Lysis Buffer | Invitrogen | 00-5333-54 |
| Fetal Bovine Serum | Atlanta Biologicals | S11150 |
| Human Serum Type AB | Fisher BioReagents | BP2525-100 |
| CountBrightTM Absolute Counting Beads | ThermoFisher | C36950 |
| PFA | Electron Microscopy Solutions | 15710 |
| EDTA Vacutainer | Fisher Scientific | 02-657-32 |
| ACD Vacutainer | Fisher Scientific | 02-684-26 |
| Heparin Vacutainer | Fisher Scientific | 367874 |
| RBC depletion Beads | Stem Cell Technologies | 18170 |
| Annexin V Apoptosis Detection Kit with PI | Biolegend | 640932 |
| BD CompBead Plus | BD Biosciences | 560497 |
| Formic Acid | Millipore Sigma | 64-18-6 |
| Sodium Chloride | Fisher Scientific | S270-10 |
| Sodium Carbonate | Alfa Aesar | 497-19-8 |
| Sodium Sulfate | Alfa Aesar | 7757-82-6 |
| Sodium Azide | Sigma-Aldrich | S8032 |
| Ammonium Chloride | Acros Organics | 12125-02-9 |
| Potassium Bicarbonate | Fisher Scientific | 298-14-6 |
Supplementary Table S2: Reagents.
